# Health-Related Quality of Life, Anxiety, and Self-Image in Young Patients With Crohn’s Disease and Ulcerative Colitis

**DOI:** 10.1097/PG9.0000000000000287

**Published:** 2023-02-02

**Authors:** Ken Lund, Torben Knudsen, Jens Kjeldsen, Rasmus Gaardskær Nielsen, Bente Mertz Nørgård

**Affiliations:** From the * Center for Clinical Epidemiology, Odense University Hospital, Odense, Denmark; † Research Unit of Clinical Epidemiology, Department of Clinical Research, University of Southern Denmark, Odense, Denmark; ‡ Department of Medicine, Hospital of Southwest Jutland, Esbjerg, Denmark; § Department of Regional Health Science, University of Southern Denmark, Esbjerg, Denmark; ‖ Department of Medical Gastroenterology S, Odense University Hospital, Odense, Denmark; ¶ Research Unit of Medical Gastroenterology, Department of Clinical Research, University of Southern Denmark, Odense, Denmark; # Hans Christian Andersen Children’s Hospital, Odense University Hospital, Odense, Denmark; ** Research Unit of Pediatrics, Department of Clinical Research, University of Southern Denmark, Odense, Denmark.

**Keywords:** inflammatory bowel disease, pediatrics, quality of life, anxiety, self-image

## Abstract

We examined health-related quality of life, anxiety, and self-image in patients aged 10–20 years with Crohn’s disease (CD) and ulcerative colitis (UC) in remission. These areas are key concerns in clinical care. We used the IMPACT-III for health-related quality of life and The Beck Youth Inventory-II for anxiety and self-image. Linear regression models were used to compare CD to UC. We included 67 patients, 44 (66%) with CD and 23 (34%) with UC. The mean score for IMPACT-III, anxiety, and self-image for CD versus UC was 78 (±SD: 13) versus 78 (±SD: 15), 44 (±SD: 9) versus 45 (±SD: 8), and 10 (±SD: 9) versus 9 (±SD: 6), respectively. We found no difference between CD and UC. Despite remission, we found an elevated score of anxiety and a low score of self-image. When evaluating mental health status, a varied approach may be beneficial for researchers.

**What Is Known**

-  Patients with Crohn’s disease may experience an overall lower health-related quality of life than ulcerative colitis.
-  Anxiety is prevalent in young patients with inflammatory bowel disease (IBD) and may be associated with physical symptoms.
-  Young patients with newly diagnosed IBD may experience psychological stress due to lack of effect of treatment.
-  Self-image is an important parameter for young patients with IBD.
-  Youth is a time in life where self-image becomes increasingly important, and body image is seldom considered as an important aspect of care in patients with IBD.

**What Is New**

-  We examined health-related quality of life using the IMPACT-III questionnaire, and anxiety and self-image using the Beck Youth Inventory-II questionnaires.
-  Despite patients in remission, we found a high score of anxiety and a low score of self-image but a high score of health-related quality of life.
-  We found no difference between patients with Crohn’s disease and ulcerative colitis.
-  We found that male had a higher IMPACT-III score than females, and females had a lower score on self-image than males.

## INTRODUCTION

In Denmark, the incidence of inflammatory bowel disease (IBD) in young patients is increasing (1,2). Health-related quality of life (HRQOL) is a key concern in IBD because of the unpredictable nature of flares and the life-long chronic illness (2).

HRQOL is a composite measure that covers the patient’s subjective perception of physical, psychological, and social well-being (2). In young patients with IBD, the disease-specific questionnaire IMPACT-III is used for measuring HRQOL (3,4). IMPACT-III is available in Danish, but the Danish version is not yet validated (5). Young patients with IBD generally have a lower HRQOL than healthy individuals, and it is known that factors such as increased disease activity, long disease duration, and treatment may predict lower HRQOL (2,6). Furthermore, patients with Crohn’s disease (CD) may have lower HRQOL scores than patients with ulcerative colitis (UC) (7). IMPACT-III is a complex metric with various domains, and it may not account for all aspects of psychological well-being, for example, anxiety and self-image. Younger patients with IBD may experience anxiety and an altered self-image because they are facing a life-long disease with the risk of a stoma. The Danish version of the Beck Youth Inventories-II includes 5 self-reported measures in total, and we focused on the subscales of anxiety and self-image (8). The Beck Youth Inventory has been validated in a Danish setting and used in a clinical study (9,10). The current knowledge on disease-specific HRQOL, anxiety, and self-image is limited as only a few studies have used various evaluation tools in the same population of young patients with IBD.

In patients, aged 10–20 years, with IBD in clinical remission, we examined disease-specific HRQOL using IMPACT-III, and examined anxiety and self-image using The Beck Youth Inventory-II comparing patients with CD with patients with UC. We did not include a healthy control group because we focused on IBD-specific HRQOL.

## METHODS

In a cross-sectional study, we collected information on demographic characteristics, pharmacological treatment, HRQOL, self-image, and anxiety in young patients (10–20 years) with IBD and a minimum disease duration of 1 year. A total of 67 patients in remission participated from July 1, 2018, to July 1, 2019. The patients were recruited from 4 hospital-based gastroenterological clinics. We included only patients in clinical remission. Remission was defined by a combination of fecal calprotectin (standard measure for all outpatient visits available from the medical record) below 200 µg/mg and an assessment of clinical remission by the patient’s physicians. We excluded the patients if the physician in charge assessed the patient or the family situation to be too stressful to participate in answering the questionnaires. We electronically forwarded the Danish IMPACT-III questionnaire to evaluate disease-specific HRQOL and the Danish Beck Youth Inventory-II questionnaire on anxiety and self-image (3,8). The IMPACT-III includes 35 items with a 5-point Likert scale and was linearly transformed to a 0–100 score. The higher score, the higher quality of life by convention (11). The Beck Youth Inventory-II questionnaire includes 20 items with a 4-point Likert scale with a maximum of 60 points for anxiety and self-image. The anxiety questionnaire covers areas of participation in social activities and health concerns associated with anxiety (8). The higher anxiety score, the worse. The self-image questionnaire covers areas of competence, energy, and positive self-esteem (8). The lower self-image score is the worst. We used a Reseach Eletronic Data CAPute data management platform database hosted at The University of Southern Denmark for data collection. Patients and their legal guardians gave written consent. Studies using questionnaires do not require ethical approval by The Regional Ethics Committee of Southern Denmark according to Danish law. The handling of data was approved by The Region of Southern Denmark Data Protection Agency (j.no. 2017-17-8498).

We used descriptive statistics with frequencies, percentages, means, and SD for normally distributed data. We provided a mean plot on HRQOL, anxiety, and self-image. We compared patients with CD to patients with UC on the measure of interest using linear regression models. We applied an unadjusted model and an adjusted model taking into account sex, age, and disease duration. We used complete cases with no missing data in the analyses. The residuals were used to evaluate normality, variation, and linearity. Further, the mean scores were stratified by disease, sex, and age groups. We used a *t* test with unequal variance to compare mean scores, and between age categories, a 1-way analysis of variance was applied.

We used multiple imputations on missing data from the questionnaires in a subanalysis. We used multivariate normal distribution as the covariates were normally distributed. We imputed 10 datasets, and for all the measures, the proportion of missing data was 20.9% (14/67 patients). The score of interest, sex, age, and disease duration was included in the model for each of the measures.

## RESULTS

The study population included 67 patients with IBD. Fifty-three (79%) had complete data on HRQOL, anxiety, and self-image. Table 1 shows the demographics of the study population.

**TABLE 1.** Characteristics of 67 pediatric and adolescent patients with inflammatory bowel disease

Characteristics	Crohn’s disease, N = 44	Ulcerative colitis, N = 23
	n (%)	n (%)
Sex		
Male	21 (47.7)	11 (47.8)
Female	23 (52.3)	12 (52.2)
Age		
Years at diagnosis, mean (SD)	13.15 ± 3.61	13.41 ± 3.53
Years at inclusion, mean (SD)	16.18 ± 2.99	16.43 ± 2.33
Age categories		
10–13	10 (22.7)	<5* (—)
14–17	15 (34.1)	12 (52.2)
18–20	19 (43.2)	7 (30.4)
Disease duration		
Years, mean (SD)	3.56 ± 2.39	3.69 ± 3.04
Disease biomarkers		
Fecal calprotectin (µg/mg), mean (SD)	68.48 ± 57.41	45.87 ± 45.13
C-reactive protein (mg/L), mean (SD)	3.02 ± 5.75	3.95 ± 14.55
Pharmacologic treatment		
Aminosalicylic acid	<5* (—)	8 (34.8)
Azathioprine or mercaptopurine	9 (20.5)	6 (26.1)
Corticosteroids	0 (0)	0 (0.0)
Biologics (infliximab, adalimumab, vedolizumab)	20 (45.5)	5 (21.7)
Combination therapy with biologics and immunosuppressives	9 (20.5)	<5* (—)
Other noncategorized drugs	<5* (—)	<5* (—)
IMPACT-III—Quality of life		
Completed	36 (81.8)	17 (73.9)
Incomplete	8 (18.2)	6 (26.1)
The Beck Youth Inventory—Anxiety and Self-image		
Completed	36 (81.8)	17 (73.9)
Incomplete	8 (18.2)	6 (26.1)

*Censored because numbers lower than 5 are not allowed to be reported according to Danish law.

**FIGURE 1.**
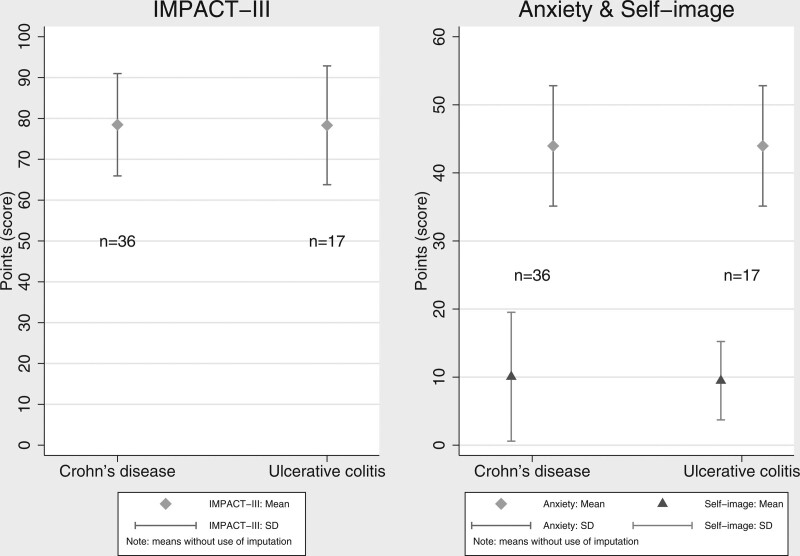
The mean and SD plot of the total IMPACT-III score (the higher, the better, 0–100 after linear transformation), anxiety score (the lower, the better), and self-image score (the higher, the better) for patients with Crohn’s disease and ulcerative colitis.

Overall, the mean score for IMPACT-III was 78 (±SD: 13), for anxiety 44 (±SD 9), and for self-image 10 (±SD 8) for the study population. The mean anxiety score was at the high end of the third quartile, 30–45 points, and the self-image score was in the lowest quartile, 0–15 points. Figure 1 shows the mean and confidence interval (CI) for the IMPACT-III score, the anxiety score, and the self-image score stratified by type of disease.

The total mean score for IMPACT-III, anxiety, and self-image for CD versus UC was 78 (±SD 13) versus 78 (±SD 15), 44 (±SD 9) versus 45 (±SD 8), and 10 (±SD 9) versus 9 (±SD 6), respectively. Comparing CD to UC, the unadjusted and adjusted difference for the IMPACT-III scores was 0.13 points (95% CI, –7.66 to 7.99) and 0.74 points (95% CI, –6.83 to 8.32), for anxiety score –1.27 points (95% CI, –6.53 to 3.99) and –1.14 points (95% CI, –6.31 to 4.03), for self-image score 0.58 points (95% CI, –4.42 to 5.60) and –0.55 points (95% CI, –5.08 to 3.98), respectively. In the subanalysis using multiple imputations to patients with missing data on the outcomes, we compared CD to UC, and this did not change the conclusion.

In an unadjusted sub-analysis stratified on sex, we observed a statistically significant difference in the IMPACT-III score of 8.4 (95% CI, 1.87–14.99) points higher for males than females, and the self-image score of 7.5 (95% CI, –11.3 to –3.75) lower for females than males. We did not observe a statistically significant difference between sex in the anxiety score. In an unadjusted analysis stratified on age categories (10–13, 14–17, 18–20 years of age), we did not observe any statistically significant variance in the outcomes.

## DISCUSSION

In patients in remission, we measured IBD-specific HRQOL with IMPACT-III; the HRQOL was high indicating overall high quality of life. However, the psychological well-being was different in these young IBD patients when we assessed the level of anxiety and self-image. Here we observed an elevated level of anxiety and a low level of self-image on scales ranging from 0 to 60. We did not find a statistically significant difference between CD and UC. We found that males had a statistically significant higher IMPACT-III score than females, and females had a lower self-image score than males.

The preservation of a high HRQOL is an important parameter in the treatment of IBD. In a review, Touma et al (6) found that disease activity is the most important determinant for HRQOL in young patients with CD. This concurs with our results of high IMPACT-III scores in patients in remission. We observed no difference in HRQOL between patients with CD and UC in line with a previous study (7). Contrary, Silva et al (12) have reported a difference in the HRQOL score between CD and UC with mild to moderate disease activity. Furthermore, the female sex has been reported as a predictor of low HRQOL, analogous to our result (12). We did observe that males had a higher IMPACT-III score than females.

The elevated level of anxiety in our study corresponds with reports that young patients with IBD may have an increased risk of anxiety (13,14). Our study used the Beck Youth Inventory-II, developed for screening for anxiety, but which is not disease-specific to IBD and cannot be used for diagnosing anxiety. Thus, we do not know the proportion of anxiety in our study population. Others have reported a proportion of around 13% for anxiety using different questionnaires in the IBD population (13,14). We were surprised to find a high score on HRQOL and at the same time, a high score on anxiety and a low score on self-image. Several explanations could apply to our findings. One reason could simply be that different evaluation tools measure different domains. For example, HRQOL might be relatively high, but the patients may still be anxious about the disease activity, future flares and surgeries that may influence their self-image captured in these specific questionnaires. Importantly, we found that females had lower scores on self-image than males, and this points towards a need to discuss such issues related to self-image in clinical care. Thus, the knowledge of anxiety and self-image is still limited, new scales have become available, for example, The IBD-specific Anxiety Scale (15). The IBD-specific Anxiety Scale was developed at the same time we performed this study. In the future, studies on HRQOL could apply the IMPACT-III in the IBD population, studies on disease-specific anxiety may well use the IBD-specific Anxiety Scale, and evaluation of IBD-specific self-image may warrant new instruments as only generic tools exist.

The main strength of the study is the use of valid tools to assess HRQOL, anxiety, and self-image (3,8). This reduces the risk of measurement bias and choosing an appropriate valid tool for measuring disease-specific HRQOL, disease-specific anxiety, and disease-specific self-image is important if available. This study has limitations. We were not able to register the number of patients refusing to take part in the study, and selection bias may be present when conducting a cross-sectional study. Yet, we have no reason to believe that selection bias is of major concern as we approached patients in remission. The limited number of patients may influence our findings by variability, and our results should be interpreted with this in mind. We did not carry out a power calculation before the initiation of the study because such a calculation would be hampered by the fact that the minimal clinically important difference of IMPACT-III is not yet established.

In conclusion, the level of HRQOL was high in young Danish patients (10–20 years) with IBD in remission, but we did observe high scores on anxiety and low scores on self-image. We did not find a statistically significant difference between patients with CD and UC. Future research on psychological well-being might well focus on patients with different states of disease activity. In addition, studies may explore differences between sex and other subgroups who might benefit from personalized clinical care from interdisciplinary teams that include psychologist.

## ACKNOWLEDGMENTS

We thank all the dedicated study coordinators for recruitment, inclusion, and collecting data at The Hans Christian Andersen Children’s Hospital at Odense University Hospital: Mette Thurøe, RN, Lene Wistrup Gerup, RN, and The Pediatric Department at Hospital Little Belt: Nanna Lind Simonsen, RN, Gitte Ozen Deinbek, RN, and The Department of Medicine at The Hospital of Southwest Jutland: Lene Uldall Thorup, RN, Charlotte Riddersholm, RN, and The Department of Medical Gastroenterology S at Odense University Hospital, Anne Berg, RN. The data storage and environment were hosted by OPEN, Open Patient data Explorative Network, Odense University Hospital, Region of Southern Denmark.
